# Machine learning for the detection of early immunological markers as predictors of multi-organ dysfunction

**DOI:** 10.1038/s41597-019-0337-6

**Published:** 2019-12-19

**Authors:** Laura Bravo-Merodio, Animesh Acharjee, Jon Hazeldine, Conor Bentley, Mark Foster, Georgios V. Gkoutos, Janet M. Lord

**Affiliations:** 1 Institute of Cancer and Genomic Sciences, Centre for Computational Biology, University of Birmingham, Birmingham, B15 2TT UK; 2 Institute of Translational Medicine, University of Birmingham, Birmingham, B15 2TT UK; 3 NIHR Surgical Reconstruction and Microbiology Research Centre, University Hospital Birmingham, Birmingham, B15 2WB UK; 4 MRC-Arthritis Research UK Centre for Musculoskeletal Ageing Research, Institute of Inflammation and Ageing, Birmingham University Medical School, Birmingham, B15 2TT UK; 5 Royal Centre for Defence Medicine, Birmingham Research Park, Birmingham, B15 2SQ UK; 6 MRC Health Data Research UK (HDR UK), Birmingham, UK; 7 NIHR Experimental Cancer Medicine Centre, B15 2TT Birmingham, UK; 8 NIHR Biomedical Research Centre, University Hospital Birmingham, Birmingham, B15 2WB UK

**Keywords:** Prognostic markers, Computational models, Predictive markers

## Abstract

The immune response to major trauma has been analysed mainly within post-hospital admission settings where the inflammatory response is already underway and the early drivers of clinical outcome cannot be readily determined. Thus, there is a need to better understand the immediate immune response to injury and how this might influence important patient outcomes such as multi-organ dysfunction syndrome (MODS). In this study, we have assessed the immune response to trauma in 61 patients at three different post-injury time points (ultra-early (<=1 h), 4–12 h, 48–72 h) and analysed relationships with the development of MODS. We developed a pipeline using Absolute Shrinkage and Selection Operator and Elastic Net feature selection methods that were able to identify 3 physiological features (decrease in neutrophil CD62L and CD63 expression and monocyte CD63 expression and frequency) as possible biomarkers for MODS development. After univariate and multivariate analysis for each feature alongside a stability analysis, the addition of these 3 markers to standard clinical trauma injury severity scores yields a Generalized Liner Model (GLM) with an average Area Under the Curve value of 0.92 ± 0.06. This performance provides an 8% improvement over the Probability of Survival (PS14) outcome measure and a 13% improvement over the New Injury Severity Score (NISS) for identifying patients at risk of MODS.

## Introduction

Trauma is responsible for the deaths of 4.9 million people worldwide according to the latest World Health Organization (WHO) reports^1^, with two thirds of patients dying as a result of sequelae other than the immediate injury and haemorrhage^2^. Secondary complications such as acute respiratory distress syndrome (ARDS), nosocomial infections, sepsis or multi-organ dysfunction syndrome (MODS) with multi organ failure (MOF) at the end of the spectrum, have therefore become increasingly significant, creating an urgent need to develop novel approaches by which to identify patients at risk. MODS, a progressive failure of several independent organ systems, occurs in 30% to 40% of trauma patients^3^, with nearly 50% of those in the Intensive Care Unit (ICU) dying as a result^4^.

Given its complexity, the underlying biological processes driving MODS have not yet been completely elucidated, although an exacerbated and dysregulated inflammatory response that impairs the body’s own healing mechanisms has been identified as a key driver of MODS development^5–7^. After a severe injury tissue damage and ischaemia lead to the release of inflammatory mediators locally that drive a pronounced systemic pro-inflammatory response (SIRS) that can promote further damage. In compensation, an anti-inflammatory response (CARS)^8^ occurs concomitantly^9^ and drives homeostasis restoration and tissue repair mechanisms. However, when exacerbated, a pronounced and extended anti-inflammatory signature can drive a state of immune suppression, leaving injured patients with increased susceptibility to secondary infections and sepsis^2^. Examining the balance between SIRS and CARS is therefore essential to understand possible outcomes for trauma patients^9^. For instance, a higher serum level of the pro-inflammatory cytokine interferon-γ (IFN-γ)^5^ and a raised frequency of natural killer T cells^9^ have been shown to be associated with MODS development^9^. Impaired bactericidal function is also seen after trauma and is associated with MODS and sepsis, possibly explained by the increase in immature neutrophils seen after trauma^9,10^. Moreover, activated platelets and neutrophil extracellular trap (NET) generation are also associated with organ dysfunction^10^.

In the *Golden Hour* study^9^, we aim to characterise the immune signature of patients during the very early response to trauma to better understand and predict their later development of adverse outcomes such as MODS and sepsis. Given the abrupt nature of a traumatic injury, knowledge of the physiological response after trauma has largely been based on post-admission to hospital blood samples. However recent studies^9–11^ have suggested that by then the body’s immune response might already be set towards a helpful or dysregulated response and subsequent positive or negative patient outcomes. Therefore, the predictive value of immune information immediately after severe injury (before 2 hours) can help provide optimal care for patients, encouraging interventions such as early total care, damage control orthopaedic surgery (DOC) or guiding immunomodulatory therapies^12,13^. Moreover, the immediate immune signature has been shown to hold predictive information for outcomes such as MODS and mortality unaccounted for in later time points^6^.

In the previous report of our data^9^, relatively simple statistical analysis and modelling was performed. Here extended analysis with approaches stemming from the field of Artificial Intelligence (AI) and in particular Machine Learning (ML), have been performed. Specifically, we have addressed the inadequacies of the current clinical scoring systems used for the assessment of trauma severity, namely New Injury Severity Score (NISS) and Probability of Survival (PS14). They are the main outcome measurements evaluated in TARN (the Trauma Audit and Research Network) and have both shown a high correlation with mortality and length of stay and act as the general predictors for outcomes such as MODS development^14^ and sepsis, irrespective of the underlying biological differences of the outcomes. NISS is based on the sum of squares of the Abbreviated Injury Scale (AIS) scores of a patient’s injuries and hence is limited to anatomical information only, whereas PS14 is modelled based on age, gender, Injury Severity Score (ISS), pre-existing medical conditions, patient outcome (up to 30 days)^15^ and Glasgow Coma Score (GCS)^16^.

Our approach attempts to incorporate immunological information in order to improve our ability to objectively assess a patient’s risk of MODS. Previous studies have shown the predictive ability of particular inflammatory effectors such as cell free DNA (cfDNA), mitochondrial DNA (mtDNA) or high mobility box 1 protein (HMGB1)^17,18^, present as a result of cell debris and necrosis associated with traumatic injuries. We believe that, based on the novelty of the study, outcomes resulting from the analysis of such early events after trauma, are of the utmost importance, and can assist in the development of new strategies to better stratify and treat patients.

Some recent examples of machine learning algorithms applied to predict pathology development in patients include one study^19^, which created a 16 variable Random Forest model with both clinical unstructured and structured data to predict hand, foot and mouth disease (HFMD). This approach improved the current clinical scoring system (AUC: 0.91) and is now being applied in practice. Furthermore, a similar approach to the one proposed here has been developed for early detection of pre-eclampsia involving the analysis of small non-coding RNAs (ncRNAs), with an AUC value of 0.86 generated after feature selection^20^. For other examples of dysregulated immune responses, namely sepsis^21^ and septic shock^22^ approaches that utilised data resulting from biomarker studies and routinely collected data held in electronic medical records yielded AUC values of 0.75 and 0.83 respectively. Moreover, three blood protein biomarkers found through various machine learning approaches have been used in the diagnosis of mild traumatic brain injury (mTBI)^23^. Although genomic information has shown an incredible predictive performance for patient outcomes^5^, due to the long assay time and cost, genomics is not practical in the trauma setting. We have thus decided to complement previous work by applying our framework to recently published transcriptomic data acquired at similar time frames, namely within 2 hours of injury, in major trauma patients^6^.

Here, we aimed to assess the predictive value of early immunological events after trauma (<1 h, 4–12 h, 48–72 h) as a diagnostic tool for MODS development. To do so, we have developed a framework consisting of both feature selection and performance evaluation. The selected features that will come out from the pipeline can be assessed as candidate biomarkers for the condition.

## Results

The immune and inflammatory response to trauma was analysed in 89 adult trauma patients (mean age 41 years, with an interquartile range (IQ) of 18-35-90 with 75 (84.3%) males) at three different time points (<1 h, 4–12 h, 48–72 h)^9^. From these 89 patients, 61 were selected for a more thorough analysis given their complete datasets and availability of main outcome scoring assessments. Patient’s NISS score has a mean of 37.2 with an IQ range of 9-34-75 and the Population Survival (PS14) value has a mean of 81.73 with an IQ range of 19.21-95.20-99.85. All the data sets contain information related to both immunological and clinical parameters with the immunological variables belonging to the following categories: neutrophil functional analysis (response to formyl-methionine-leucine-phenylalanine (fMLF)), monocyte response to lipopolysaccharides (LPS) stimulation, haematological, cfDNA analysis and serum analysis (cytokines and cortisol). A more detailed description of all the parameters analysed can be found in the paper by Hazeldine *et al*.^9^ and in Supplementary Fig. 1.

**Fig. 1:**
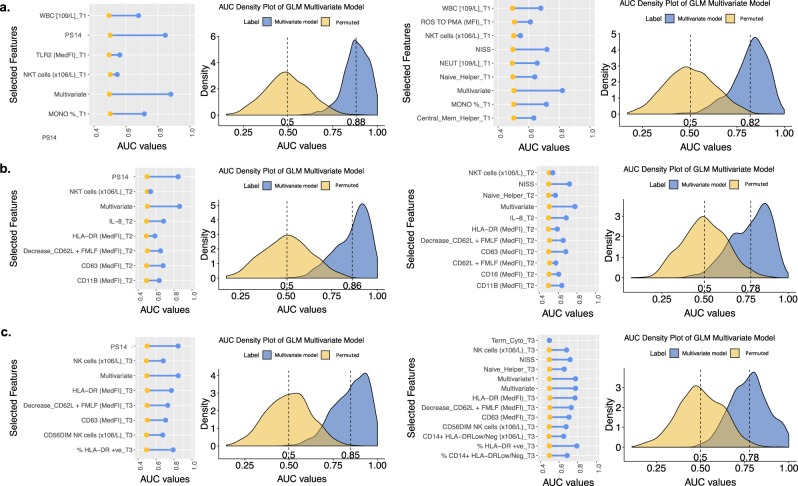
Univariate and multivariate performance (AUC values) of the selected features for the three different time points with PS14 (left) and NISS (right). The AUC performance values for each time point (**a**) <1 h, (**b**) 4–12 h, (**c**) 48–72 h are a result of 1000 GLM models of both permuted (yellow) and normal/real data (blue). On the left hand side of each figure a dumbell plot for each of the selected features per time point and the combination of all under “Multivariate” is presented. For each of these features, a univariate GLM model was built, with the average AUC values plotted. Here we present the selected features for each time point, and so those that correspond to the first quantile of frequencies of the 100 sparse models created in the LASSO and EN feature selection models (Methods: Fig. 5). On the right hand side, a density plot of the AUC values of the 1000 permuted and non permuted GLM models of the selected features per time point is depicted. The average AUC value is presented in bold.

For each of the study time points, two feature selection algorithms for MODS development, namely Least Absolute Shrinkage and Selection Operator (LASSO) and Elastic Net (EN) were applied. The performance of the chosen features was then analysed through multivariate, and univariate machine learning models, with generalized linear models (GLM) having the best performance. Due to the high correlation between NISS and PS14 and the fact that they aggregate complex information such as gravity of the wound to estimate the extent of trauma damage, they are the predominant selected features at all time points, therefore analysis was performed in each time point separately (Fig. 1) to uncover the other important features.

### Predictive model of MODS development

**Fig. 2:**
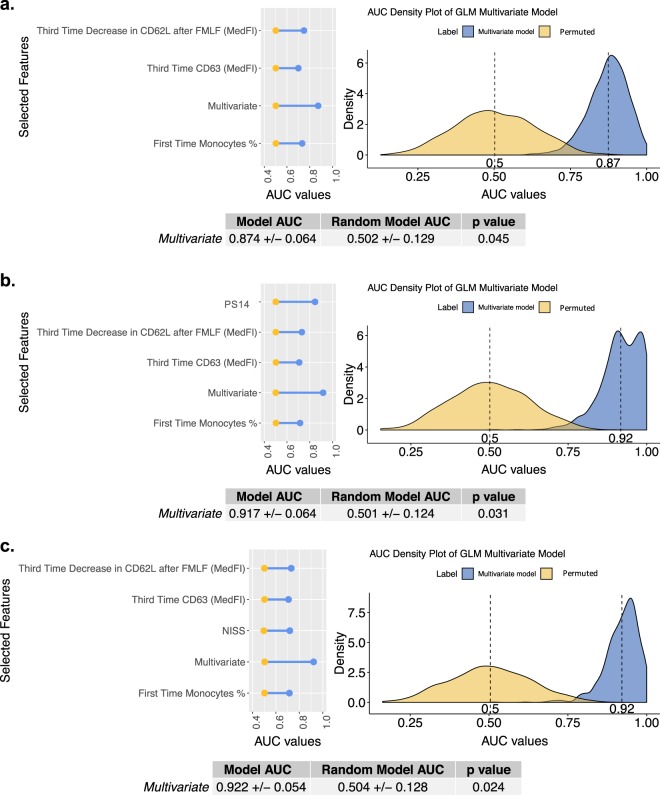
Univariate and multivariate performance (AUC values) of the selected features for the integrated time points. (**a**) Univariate GLM AUC performance values of the selected features for the integrated data set containing features at all time points. (**a**) Density plot of the AUC values of the 1000 permuted and non-permuted GLM models of the selected features together is depicted. (**b**) Density plot of the AUC values of the 1000 permuted and non-permuted GLM models of the selected features plus PS14. (**c**) Density plot of the AUC values of the 1000 permuted and non-permuted GLM models of the selected features plus NISS. A confidence interval and p-value information of the multivariate model is also included in all figures. P-value was evaluated by assessing how many times a permuted value’s AUC was above the non-permuted/real AUC mean.

We repeated the analysis using data integrated from the three different time points and applied a more stringent threshold for feature selection. The final selected features were not significantly correlated and showed both biological significance and clinical relevance given the low cost and short time taken to perform their analysis (Fig. 2). By selecting the features irrespective of time points, we are selecting the overall highest associated changes with MODS development. With this approach, a further understanding of how the response develops might be reflected in this combination of time points, with multifaceted relations between components and kinetics revealed.

This multivariate combination of the third time point neutrophil decrease in CD62L expression after fMLF treatment and CD63 expression and monocyte frequency from the first time point resulted in the best performing models. By themselves, an AUC value of 0.874 ± 0.064 was achieved. The prediction ability of the model was then further improved with the addition of NISS and PS14 clinical scores, where AUC values of 0.917 ± 0.064 AUC and 0.922 ± 0.054 were obtained respectively. All three models, as seen in Fig. 2, were statistically significant with respect to pure randomness as exemplified by permuted data. By comparing with their univariate model performance, we can further establish a 13 and 8% increase in the predictive capability of NISS and PS14 scores, an improvement which, although not statistically significant, is of clinical importance (Supplementary Fig. 2).

### Transcriptomics data analysis

In addition to the inflammatory markers, we used transcriptomics data obtained from the previous publication by Cabrera *et al*.^6^ and applied our feature selection framework to these data. The phenotypic information associated with this dataset, collected at similar ultra-early post-trauma time intervals, is consistent with the phenotypic information in our datasets. Crucially, traumatic brain injury patients were not included in either study or patients that received blood products prior to hospital admission. From the 29,385 genes analysed, AMD1, C3AR1, FLI1, GIMAP4, GPI, GPR84, HLA-A29.1, LOC643373, MYLIP, VAT1, MANBA, SRXN1 were selected based on our feature selection algorithms with respect to MODS vs non-MODS classification prediction. A Gene Ontology Enrichment Analysis^24,25^ was performed on the selected genes, with the enriched biological pathways being myeloid leukocyte mediated immunity, neutrophil activation and neutrophil degranulation. A p-value of 2.8288e^−5^ was obtained for the neutrophil degranulation pathway associated with the VAT1, GPI, C3AR1, GPR84 and MANBA genes. More information on the chosen gene’s distribution can be found in Supplementary Fig. 3.

## Discussion

MODS is a complex multifactorial syndrome which develops in 30–40% of trauma patients^3^. In this study, we aimed to improve our understanding of its biological underpinnings through the analysis of the most relevant immunological markers at an ultra-early time point after injury and their changing dynamics at different time periods. In this way we have created a framework that allows the analysis of biologically relevant features so as to inform a more effective and timely medical intervention as well as patient stratification for therapy or inclusion in clinical trials. To do so, we have assessed the predictive power of early immunological and clinical data for MODS development after injury. As a result, a combination of four features have been determined as relevant clinical biomarkers, with a classification performance of 0.92 AUC value. All four features can be measured through inexpensive and technically feasible assays.

In this study, we performed validation in two ways, one is based on computational or *in silico* validation and the other is based on clinical or expert knowledge. In the computational framework we included double cross validation as recommended in the literature to limit possible false positives and overfitting during training^26–28^. First, for feature selection, we performed a random split of the samples with 75% being training and 25% test. Further, we optimised our parameters on the 75% of the training samples by applying cross validation.

Feature selection was performed through two regularization methods: LASSO and EN. These methods were selected as they generate sparse models with ranking and selection of features, with easily interpretable frameworks and results (linear models) suitable for the clinical data being analysed^29^. During performance assessment and after a 65% training and 35% testing split of the data, a model averaging was done to estimate the error rate on multiple iterations for an unbiased estimate to investigate the stability of the potential markers. In the literature recent studies have emphasized this aspect of using double cross validation to overcome the overfitting of –omics data sets. For example Acharjee *et al*. used a similar strategy on a metabolomics data set^26^, Filzmoser *et al*. applied this on near infrared (NIR) data^27^, Christin *et al*. performed it on proteomics and metabolomics data sets^28^. Regarding clinical validation, we included immunologists and clinicians’ expert opinions to the biomarkers chosen in addition to the literature based evidence.

Hazeldine *et al*.^9^ reported that “the complex nature of the acute immune response to injury suggests that targeting one element of the immune response is unlikely to reduce immune paresis or the incidence of MODS”. Understanding and predicting MODS development thus necessitates use of a multivariate approach that is able to reproduce heterogeneous and complex interactions. Other recent scoring systems such as A Severity Characterization Of Trauma (ASCOT) combine physiological parameters such as age and systolic blood pressure^30^, and clearly demonstrate the value of physiological assessment. With the use of the current anatomically based scoring system (NISS) alone, a good MODS development predictive model can be developed with an AUC value of 0.72 (Figs. 1 and 2). But with the inclusion of data related to underlying immunological processes, an improvement of 13% with respect to NISS alone was achieved. Whilst NISS plays a vital role in the performance of a MODS prediction model (Supplementary Fig. 2), the discovery of relevant biological descriptors offers the possibility of potentially identifying clinical targets that may be used for intervention. For the future, we hope to find a marker or aggregation of markers able to substitute the complex anatomical information reflected in NISS and PS14 scores and so yield an objective and real time predictive parameter^31,32^. cfDNA and HMGB1 have been proposed as plausible substitutes^18^, though they were not selected here as the main features at any time point due to missing data. Moreover although our objective was to obtain a real time predictive parameter (i.e under 1 hour and easily available to clinicians), the features selected still allow for a prompt assay in a routine clinical lab and prediction of the patients outcome, given that MODS is assigned to those patients with a SOFA score of 6 or more in 2 consecutive days at least 48 hours post admission. Therefore, the earliest day MODS could be diagnosed would be day 4 post injury, with our algorithm able to predict this outcome at least 12 h before.

In order to further validate our results and create a generalizable model, more samples and of various origins are required. For example, since the same number of samples is required at each time point, studies similar in nature to ours, are likely to suffer from the fact that later time points are more likely to yield less samples (due to patient discharge or death etc). Therefore, the need for imputing data related to measurements from samples collected at later time points is especially high and hence the model’s prediction outcome should be critically evaluated and considered. Moreover, in our study, women were under represented (16% of patients being female). Therefore, sex differences were not corrected for which could have yielded different outcomes^12^. Moreover, extra features could be of interest too, such as information on the adaptive immune cell frequency or function or metabolite levels^33^. For example, albumin and lactate are two biomarkers of special interest given that they have been identified as having a significant correlation with the development of early MODS^34–36^. However, measurements of albumin were not obtained in our study and lactate has not been identified as a key predictor of MODS in our studies. The latter could be attributed to the small number of patient samples that lactate measurements were available for, with the data shown in Supplementary Fig. 4.

**Fig. 3:**
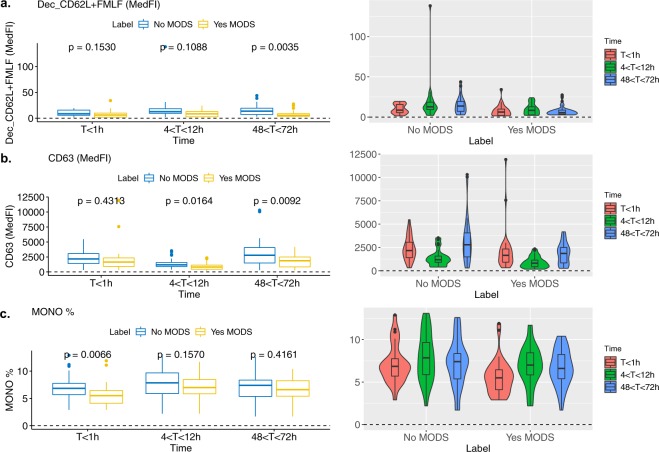
Visualisation of the non-normalized data distribution of the selected features after all time points data integration. (**a**) Decrease in neutrophil CD62L (L-selectin) after stimulation with fMLF (48–72 h). (**b**) Neutrophil CD63 expression (48–72 h) and (**c**). (**a**) Monocyte percentage plots for all patients (<1 h). Right: Violin-boxplot exhibiting change of marker through time for MODS and non-MODS patients. Left: Boxplot with p-value comparison following unpaired t-test between time points (<1 h, 4–12 h, 48–72 h).

All three features selected by our approach concern neutrophil function or frequency changes (Fig. 3). This is unsurprising as it is well understood that traumatic injury leads to marked alterations in the phenotype, function and life-span of circulating neutrophils with significant changes in the composition of the circulating neutrophil pool^5^. L-selectin (CD62L) is a receptor that facilitates tethering of neutrophils to the endothelium in inflammation and it has been shown to be significantly reduced on circulating neutrophils following severe trauma^37,38^. In our study, the bacterial tripeptide fMLF was used as a neutrophil stimulator *ex vivo*. Upon exposure to fMLF neutrophils would normally shed CD62L from their cell surface^39^, with increased levels of its soluble form present in the plasma as a result. In this study, shedding was less prominent in MODS patients than in non-MODs at all time points (Fig. 3a) suggesting a level of immunoparesis. A similar phenomenon has been previously reported in other receptors such as fMLF-induced expression of FcγRII (CD32)^40^. Reduced expression of CD63, a marker of primary granule exocytosis on the surface of neutrophils, is a well known marker for neutrophil activation. Primary granules or azurophils contain proteolytic enzymes such as elastase, a chief contributor to tissue damage in trauma patients^41^. Significantly, at time point three (48 to72h), patients who developed MODS had lower CD63 expression on their neutrophils when compared to patients who did not develop MODS (Fig. 3b). We hypothesize that this apparently less active peripheral pool of neutrophils in patients who have a worse outcome is due to those activated CD63 expressing neutrophils migrating into tissues/organs where they would cause damage and potentially contribute to the pathogenesis of MODS. Finally, it is likely that the significantly lower monocyte percentage associated with the development of MODS in the first time point (<1 h) (Fig. 3c) is simply a consequence of the higher frequency of neutrophils and immature granulocytes. In particular, medium-chain fatty acid receptor GPR84 and vesicle amine transport protein-1 (VAT-1), are both relevant to neutrophil function and the inflammatory response by regulating the oxidative burst^42^ and the response to fMLF respectively^43^. This is of interest given the tight relationship with the CD62L response to fMLF in our models for MODS development and this requires further follow up.

The transcriptomics data were used as a means of complementing our immunological results and so provide a more insightful biological understanding of the early trauma response. As explained above, finding data comparable to our experimental design is difficult given its novelty but the transcriptomics data from Cabrera *et al*., 2017 allowed integration given similar sampling times (<2 h, 24 h, 72 h and <1 h, 4–12 h, 48–72 h), and the use of MODs as an outcome variable (with similar criteria). We envisage the merging of different data types (immune and transcriptomics) will allow us to better understand MODS in our analysis. Support for this comes from our gene enrichment analysis where neutrophil activation and degranulation came out as important in the transcriptomic data, in concordance with the immune markers associated with neutrophils that we found in our immune phenotype data.

To conclude, we present here a generalizable and automated framework that allows a reliable model with the yielding of a promising and encouraging preliminary result in MODS development prediction however, as a follow up study an independent validation cohort is required. Our approach not only improves the performance of the current clinical scoring systems PS14 by 8% and NISS by 13% but, and perhaps more crucially, unveils possible biomarkers and mechanistic understanding of use for future prophylactic treatments for MODS.

## Methods

### Datasets

Data was obtained from 89 adult trauma patients (ISS ≥ 8) enrolled into the Brain Biomarkers After Trauma Cohort Study (NRES 13/WA/0399), a prospective observational study undertaken at the Queen Elizabeth Hospital Birmingham, UK. The development of MODS, defined by a Sequential Organ Failure Assessment (SOFA) score of 6 or more, on two or more consecutive days, at least 48 hours post-admission was the primary outcome of interest^9^. Development of MODS acted as the label for our binary classification models, where a score of 1 or 0 was assigned to patients who did or did not develop MODS respectively. The blood samples were acquired within 1 hour of injury (mean time to sample 42 minutes), 4–12 and 48–72 h post injury. The Probability of Survival (PS14) was calculated by trained staff from the Trauma and Audit Research Network (TARN). It was modelled based on age, gender, Injury Severity Score (ISS), pre-existing medical conditions via the Charlson Comorbidity index (CCI), patient outcome (up to 30 days) and Glasgow Coma Score (GCS). If GCS was missing, intubation was used instead^16^. Further information regarding patient selection, consent and blood sampling has been described elsewhere^9^.

The transcriptomics data was publicly available from github/C4TS/HyperacutePhase^6^, with raw and normalised gene expression values available under E-MTAB-5882. We obtained and applied our feature selection algorithm to the normalised data using MODS vs nonMODS as label. The normalised data, contained gene expression values for 22,895 genes from samples obtained at different time points (<2 h, 24 h and 48 h) from 36 patients. Out of the 36 patients, 16 developed MODS and 20 did not. In this case, MODS was defined through a sequential organ failure assessment (SOFA) score of ≥5 (Golden Hour project establishes ≥6) on 2 or more consecutive days, excluding the first 48 hours. Moreover, these patients had blunt trauma and an ISS score above or equal to 25, with no traumatic brain injury. Further details about the normalisation procedure can be found in Cabrera *et al*.^6^.

### Data pre-processing

**Fig. 4:**
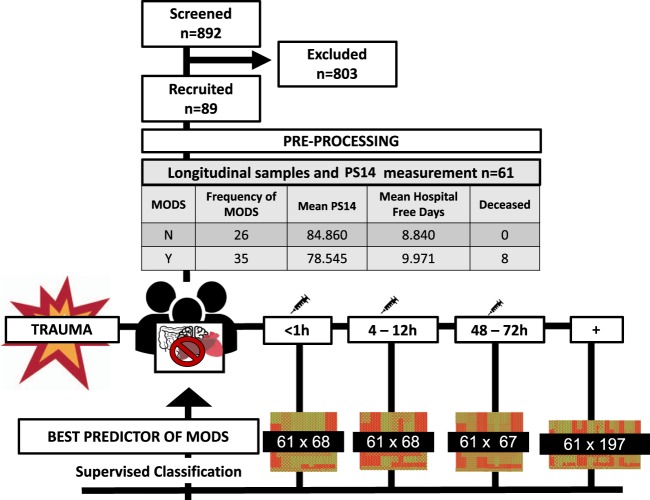
Framework and pipeline of work. Four different data sets were run through the pipeline created consisting of feature selection and machine learning performance evaluation for best predictor of multiorgan dysfunction. These four datasets had the same patients studied in a longitudinal fashion with assessment of the three different time points and their combination.

Our data pre-processing approach, essential to obtain meaningful information from down-stream statistical analysis, involved four different stages, namely data cleaning and organising, distribution of data analysis visualization, missing data imputation and treatment of outliers, and normalisation. We treated missing values (NAs) in the following manner: Data with more than 70% missing values was eliminated and the remaining data were imputed where necessary. Imputations were introduced as the minimum value found per feature, after which, the datasets corresponding to the three different time points were autoscaled (standardised) using the *scale* function available within the *norm* R package. Patients that were able to be followed up for the three time points only were included, yielding three matrices of 61 patients and 68 features for time points one and two and 61 patients and 67 features for time point 3. A diagram of the steps followed is shown in Fig. 4, with more information about the selected features in Supplementary Fig. 1. For extra analysis, all three matrices were then blended into one big data matrix of 61 patients and 197 features. We have made all the code available in our GitHub repository github/InFlamUOB/NSDTraumaMODS.

### Statistical and machine learning algorithms

Feature reduction was performed via applying two feature selection methods, Least Absolute Shrinkage and Selection Operator (LASSO) (1996)^44^ and Elastic Net (2005)^45^, two forms of regularization able to automatically select significant variables by shrinking the coefficients of unimportant predictors to zero (sparse representation). Our pipeline^29^ is composed of a variety of statistical machine learning modules described below.

### Feature selection module

In order to apply the LASSO and EN algorithms for biologically relevant feature selection, we optimised the penalty parameter associated with each of the methods in an unbiased manner. To achieve this, the pipeline divided the samples randomly into a training set composed of 75% of the total number of the samples and a test set consisting of the remaining 25% samples. A 10-fold cross validation was then applied on the training set (inner loop set) aiming to have an optimised penalty parameter that can be used in the LASSO and EN models. Mathematically, LASSO and EN models can be defined by using a single penalty function “*α*”^29,44,45^ (Eq. 1).

1 $$\frac{minimize}{\beta \in {R}^{p}}\frac{1}{2}{\Vert y-X\beta \Vert }_{2}^{2}subject\,(1-\alpha ){\Vert \beta \Vert }_{1}+\alpha {\Vert \beta \Vert }^{2}$$

For example, for a penalty parameter *α* = 1, the LASSO algorithm is applied, whereas for *α* = 0.5 Elastic Net is performed. A high-value penalty parameter allows for a more stringent selection algorithm, with more *β* coefficients reduced to 0. At α= 0, no feature selection is performed. These algorithms are implemented through the glmnet, ROC and caret packages.

### Identification of candidate biomarkers

**Fig. 5:**
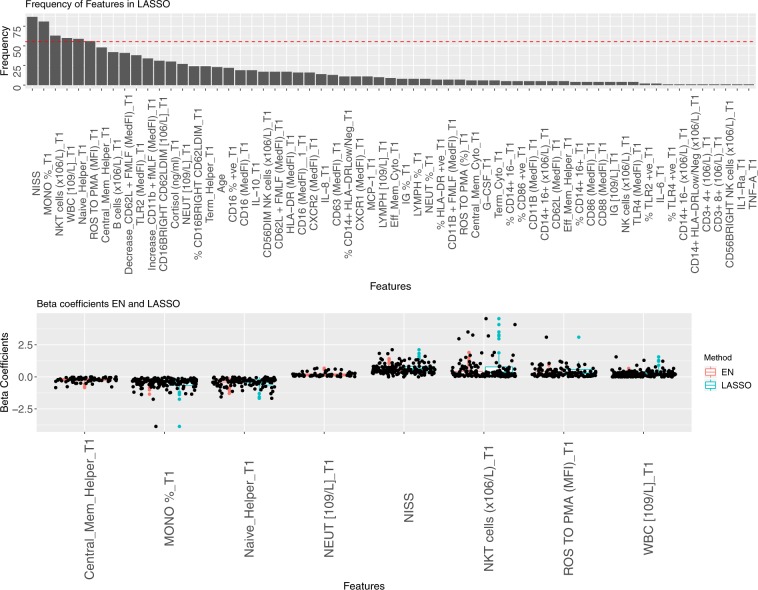
Feature selection and beta coefficients for both LASSO and EN algorithms for the first time point with NISS. (**a**) A graphical representation representing the frequency of feature appearance in the 100 models run for LASSO. Those features that appear in more than the threshold (above dotted line) are then selected. (**b**) A representation of the 100 beta coefficients found for each of the 100 models of the features selected by both algorithms (LASSO in blue and EN in orange).

Our pipeline iterated the model creation process 100 times and features that appeared more frequently than a threshold number of times (the first upper quantile) were selected (Fig. 5), as these are deemed to be the more significant for the classification model. Moreover, in order to better understand the relationship between the features selected and the outcome variable analysed, weight’s (*β* coefficients) distribution per model was displayed and a boxplot of the class differences per feature generated. These selected features are then considered as potential candidate biomarkers. In order to ascertain their validity as biomarkers, their performance is evaluated both alone and in combination.

### Performance evaluation and visualization

In order to assess the performance of the selected markers, our pipeline includes a stability analysis performed via a permutation test. This includes the randomization of the label features, resulting in incorrect sample labels for predictions and generating models with ROC AUC values showing a performance subject to the random distribution. A generalized linear model (GLM) was applied on 1000 random samplings of 65% training and 35% test sets in both real data (correct simple label) and permuted ones. ROC AUC performance results were computed for each of these models and plotted as density plots alongside their means (Figs. 1 and 2). The ROC AUC offers a graphical overview of the diagnostic ability of binary classifiers with varying thresholds.

### Software and data repositories

This pipeline was executed with four different datasets as seen in Fig. 4. At each time point the most important features were selected. A final data matrix, created through the combination of all the previous matrices together, was used to identify the most relevant features for MODS classification for the early immunological response (<1 h to 72 h). All analyses were performed in the R statistical computing (R version 3.5.2) environment. All R packages can be obtained from our project’s github repository. The necessary software dependencies are described in the README file located in the repository. All analyses can be performed on a standard PC environment with the run time increasing with larger datasets. Code is compiled and figures and key data such as selected features stored automatically for accessible future use as explained in the README file associated. Plots were generated with *geom_dumbell* and *ggplot2* functions.

### Gene ontology (GO) enrichment analysis

Enrichment analysis were performed through both Enrichr^24^ and Reactome^25^ platforms after multiple test correction.

## Supplementary information

Supplementary Information.

## Data Availability

The complete 89 patient dataset can be found in Hazeldine *et al*.^9^ and the 61 patient dataset at the three time points with which we have generated all results is available in our github repository InFlamUOB/NSDTraumaMODS^46^ and figshare^47^. All of the figures, including supplementary, have also been added to figshare (10.6084/m9.figshare.10322099).

## Code availability

The scripts used for all data analyses, implemented in R are available in the github repository github/InFlamUOB/NSDTraumaMODS^46^ and accessible through figshare too^48^.

## References

[1] Geneva World Health Organization 2018. Global Health Estimates 2016: Disease burden by Cause, Age, Sex, by Country and by Region, 2000–2016. *WHO*, http://www.who.int/healthinfo/global_burden_disease/estimates/en/ (2018).

[2] Lord JM (2014). The systemic immune response to trauma: an overview of pathophysiology and treatment. Lancet Lond. Engl..

[3] Shepherd JM, Cole E, Brohi K (2017). Contemporary Patterns of Multiple Organ Dysfunction in Trauma. Shock Augusta Ga.

[4] Mayr VD (2006). Causes of death and determinants of outcome in critically ill patients. Crit. Care.

[5] Manson, J. *et al*. Early changes within the lymphocyte population are associated with the development of multiple organ dysfunction syndrome in trauma patients. *Crit. Care* **20**, 176 (2016). 10.1186/s13054-016-1341-2 PMC4895987 27268230

[6] Cabrera CP (2017). Signatures of inflammation and impending multiple organ dysfunction in the hyperacute phase of trauma: A prospective cohort study. Plos Med..

[7] Naumann DN (2018). Endotheliopathy of Trauma is an on-Scene Phenomenon, and is Associated with Multiple Organ Dysfunction Syndrome: A Prospective Observational Study. Shock.

[8] Islam MN, Bradley BA, Ceredig R (2016). Sterile post-traumatic immunosuppression. Clin. Transl. Immunol..

[9] Hazeldine J (2017). Prehospital immune responses and development of multiple organ dysfunction syndrome following traumatic injury: A prospective cohort study. PLoS Med..

[10] Naumann DN (2017). Endotheliopathy is associated with higher levels of cell-free DNA following major trauma: A prospective observational study. Plos One.

[11] Xiao W (2011). A genomic storm in critically injured humans. J. Exp. Med..

[12] Huber-Lang M, Lambris JD, Ward PA (2018). Innate immune responses to trauma. Nat. Immunol..

[13] Spruijt NE, Visser T, Leenen LP (2010). A systematic review of randomized controlled trials exploring the effect of immunomodulative interventions on infection, organ failure, and mortality in trauma patients. Crit. Care Lond. Engl..

[14] Rendy L, Sapan HB, Kalesaran LTB (2017). Multiple organ dysfunction syndrome (MODS) prediction score in multi-trauma patients. Int. J. Surg. Open.

[15] Bouamra O (2015). Prediction modelling for trauma using comorbidity and ‘true’ 30-day outcome. Emerg. Med. J..

[16] Javali RH (2019). Comparison of Injury Severity Score, New Injury Severity Score, Revised Trauma Score and Trauma and Injury Severity Score for Mortality Prediction in Elderly Trauma Patients. Indian J. Crit. Care Med. Peer-Rev. Off. Publ. Indian Soc. Crit. Care Med..

[17] Wang H, Yang H, Czura CJ, Sama AE, Tracey KJ (2001). HMGB1 as a Late Mediator of Lethal Systemic Inflammation. Am. J. Respir. Crit. Care Med..

[18] Cohen MJ (2009). Early release of high mobility group box nuclear protein 1 after severe trauma in humans: role of injury severity and tissue hypoperfusion. Crit. Care Lond. Engl..

[19] Liu, G. *et al*. Developing a Machine Learning System for Identification of Severe Hand, Foot, and Mouth Disease from Electronic Medical Record Data. *Sci. Rep*. **7**, 16341 (2017). 10.1038/s41598-017-16521-z PMC5703994 29180702

[20] Yoffe L (2018). Early Detection of Preeclampsia Using Circulating Small non-coding. RNA. Sci. Rep..

[21] Taneja I (2017). Combining Biomarkers with EMR Data to Identify Patients in Different Phases of Sepsis. Sci. Rep..

[22] Henry KE, Hager DN, Pronovost PJ, Saria S (2015). A targeted real-time early warning score (TREWScore) for septic shock. Sci. Transl. Med..

[23] Peacock, W. F. I. *et al*. Derivation of a Three Biomarker Panel to Improve Diagnosis in Patients with Mild Traumatic Brain Injury. *Front. Neurol*. **8**, 641 (2017). 10.3389/fneur.2017.00641 PMC5714862 29250027

[24] Kuleshov MV (2016). Enrichr: a comprehensive gene set enrichment analysis web server 2016 update. Nucleic Acids Res..

[25] Fabregat A (2017). Reactome pathway analysis: a high-performance in-memory approach. BMC Bioinformatics.

[26] Acharjee, A., Finkers, H. J., Visser, R. G. F. & Maliepaard, C. A. Comparison of Regularized Regression Methods for ~Omics Data. *Metabolomics Open Access* **3**, 129 (2013).

[27] Filzmoser P, Liebmann B, Varmuza K (2009). Repeated double cross validation. J. Chemom..

[28] Christin C (2013). A Critical Assessment of Feature Selection Methods for Biomarker Discovery in Clinical Proteomics. Mol. Cell. Proteomics MCP.

[29] Bravo-Merodio L, Williams JA, Gkoutos GV, Acharjee A (2019). -Omics biomarker identification pipeline for translational medicine. J. Transl. Med..

[30] Woodford M (2014). Scoring Systems for Trauma. BMJ.

[31] Raymond, S. *et al*. Prospective Validation of a Transcriptomic Metric in Severe Trauma. *Ann. Surg*., 10.1097/SLA.0000000000003204 (2019). 10.1097/SLA.0000000000003204 PMC6656642 30688688

[32] Rittirsch D (2015). Improvement of prognostic performance in severely injured patients by integrated clinico-transcriptomics: a translational approach. Crit. Care Lond. Engl..

[33] Ferrario M (2016). Mortality prediction in patients with severe septic shock: a pilot study using a target metabolomics approach. Sci. Rep..

[34] Feng J-Y (2018). Predictors of Early Onset Multiple Organ Dysfunction in Major Burn Patients with Ventilator Support: Experience from A Mass Casualty Explosion. Sci. Rep..

[35] Wang B (2015). Correlation of lactate/albumin ratio level to organ failure and mortality in severe sepsis and septic shock. J. Crit. Care.

[36] Guyette F (2011). Prehospital serum lactate as a predictor of outcomes in trauma patients: a retrospective observational study. J. Trauma.

[37] Li H (2015). Mitochondrial damage-associated molecular patterns from fractures suppress pulmonary immune responses via formyl peptide receptors 1 and 2. J. Trauma Acute Care Surg..

[38] Drifte G, Dunn-Siegrist I, Tissières P, Pugin J (2013). Innate Immune Functions of Immature Neutrophils in Patients With Sepsis and Severe Systemic Inflammatory Response Syndrome*. Crit. Care Med..

[39] Mommsen P (2011). Regulation of L-selectin expression by trauma-relevant cytokines. Pathol. - Res. Pract..

[40] Hietbrink F, Koenderman L, Althuizen M, Leenen LPH (2009). Modulation of the innate immune response after trauma visualised by a change in functional PMN phenotype. Injury.

[41] Hashiguchi N, Chen Y, Rusu C, Hoyt DB, Junger WG (2005). Whole-Blood Assay to Measure Oxidative Burst and Degranulation of Neutrophils for Monitoring Trauma Patients. Eur. J. Trauma.

[42] Gaidarov I (2018). Embelin and its derivatives unravel the signaling, proinflammatory and antiatherogenic properties of GPR84 receptor. Pharmacol. Res..

[43] Faugaret D, Chouinard FC, Harbour D, El azreq M-A, Bourgoin SG (2011). An essential role for phospholipase D in the recruitment of vesicle amine transport protein-1 to membranes in human neutrophils. Biochem. Pharmacol..

[44] Tibshirani R (1994). Regression Shrinkage and Selection Via the Lasso. J. R. Stat. Soc. Ser. B.

[45] Zou H, Hastie T (2005). Regularization and variable selection via the elastic net. J. R. Stat. Soc. Ser. B Stat. Methodol..

[46] (2019). Zenodo.

[47] Bravo L, Acharjee A, Gkoutos VG, Lord JM, Hazeldine J (2019). figshare.

[48] Bravo L (2019). figshare.

