# Supplementary material for: Machine learning for the detection of early immunological markers as predictors of multi-organ dysfunction
Source: Sci Data. 2019 Dec 19;6:328. doi: 10.1038/s41597-019-0337-6 (PMC6923383; doi:10.1038/s41597-019-0337-6)
Supplement: Supplementary file 1 — Supplementary Information. [file 41597_2019_337_MOESM1_ESM.pdf]

Machine learning for the detection of early immunological markers as predictors of Multi-Organ Dysfunction

Laura Bravo-Merodio [1,2,*] , Animesh Acharjee [1,2,3,*#], Jon Hazeldine [3,4], Conor Bentley [3,4] , Mark Foster [3,5] , Georgios V. Gkoutos [1,2,3,6,7,8], Janet M Lord [3,4,8,]

[1]Institute of Cancer and Genomic Sciences, Centre for Computational Biology, University of Birmingham, B15 2TT, UK.
[2]Institute of Translational Medicine, University of Birmingham, B15 2TT, UK
[3] NIHR Surgical Reconstruction and Microbiology Research Centre, University Hospital Birmingham, Birmingham B15 2WB, UK.
[4] MRC-Arthritis Research UK Centre for Musculoskeletal Ageing Research, Institute of Inflammation and Ageing, Birmingham University Medical School, Birmingham B15 2TT, UK.
[5] Royal Centre for Defence Medicine, Birmingham Research Park, Birmingham B15 2SQ, UK.
[6]MRC Health Data Research UK (HDR UK)
[7]NIHR Experimental Cancer Medicine Centre, B15 2TT, Birmingham, UK
[8] NIHR Biomedical Research Centre, University Hospital Birmingham, Birmingham, B15 2WB, UK.

*These authors made equal contributions
# Corresponding Author

Supplementary Information

Content

Figures

Supplementary Fig.1 ……………………………………………………page 6,14
Supplementary Fig.2 ……………………………………………………page 7,10
Supplementary Fig.3 ……………………………………………………page 8
Supplementary Fig.4 ……………………………………………………page 10

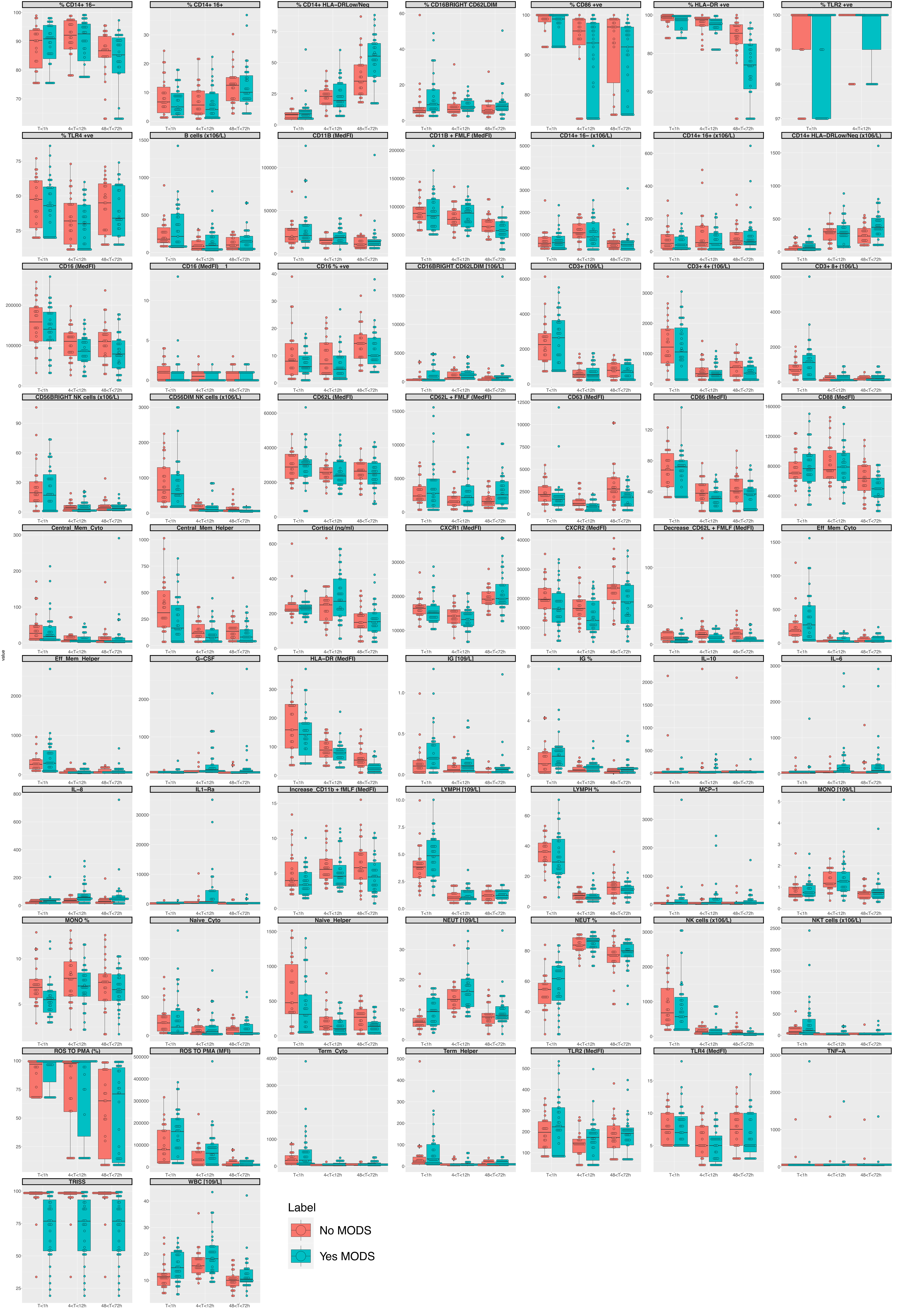

Supplementary Figure 1. **Dot boxplot of all raw variables used in the study.**

a.

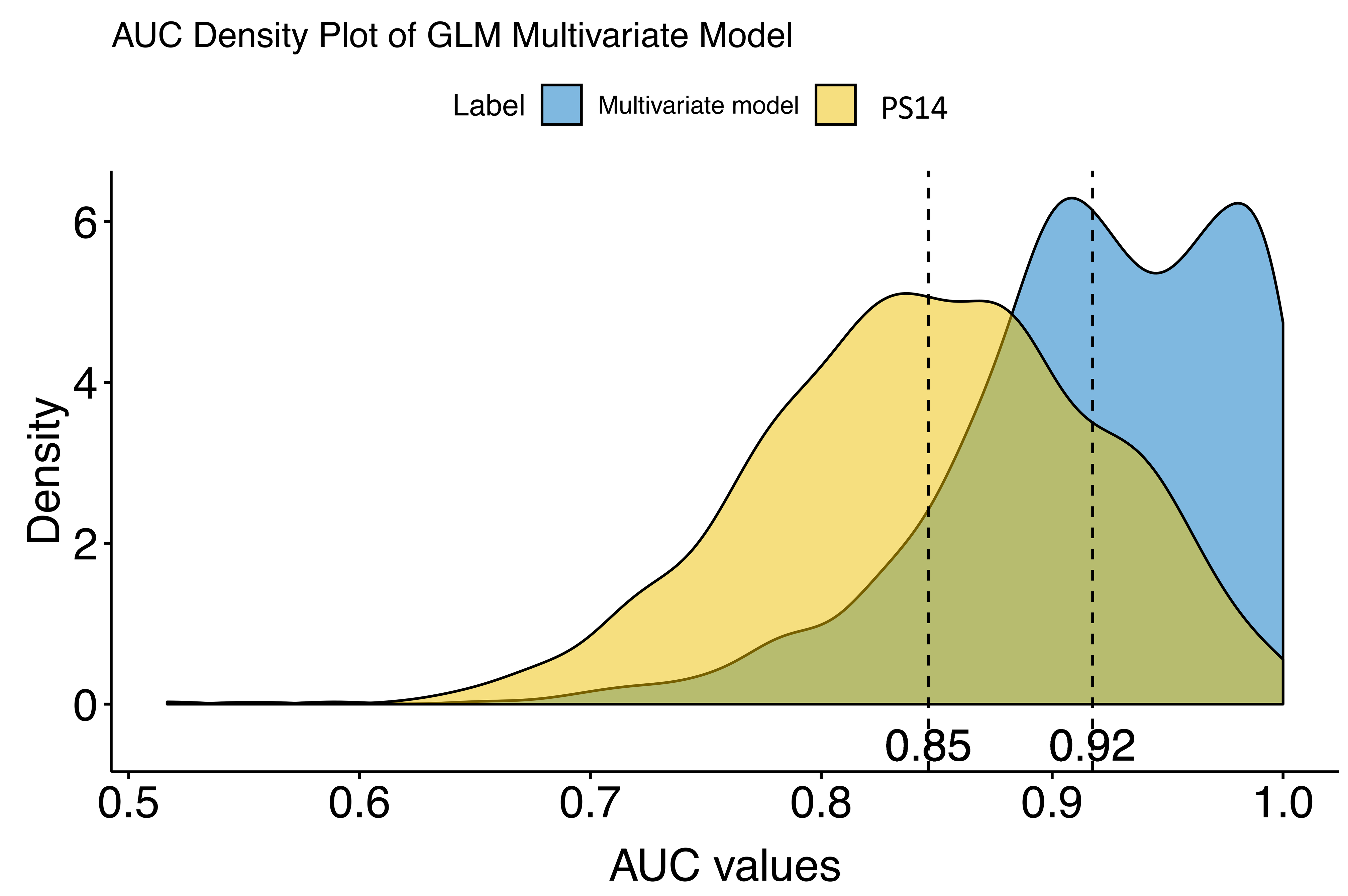

	Model AUC	Random Model AUC	p value
Multivariate	0.917 +/– 0.064	0.846 +/– 0.073	0.175

b.

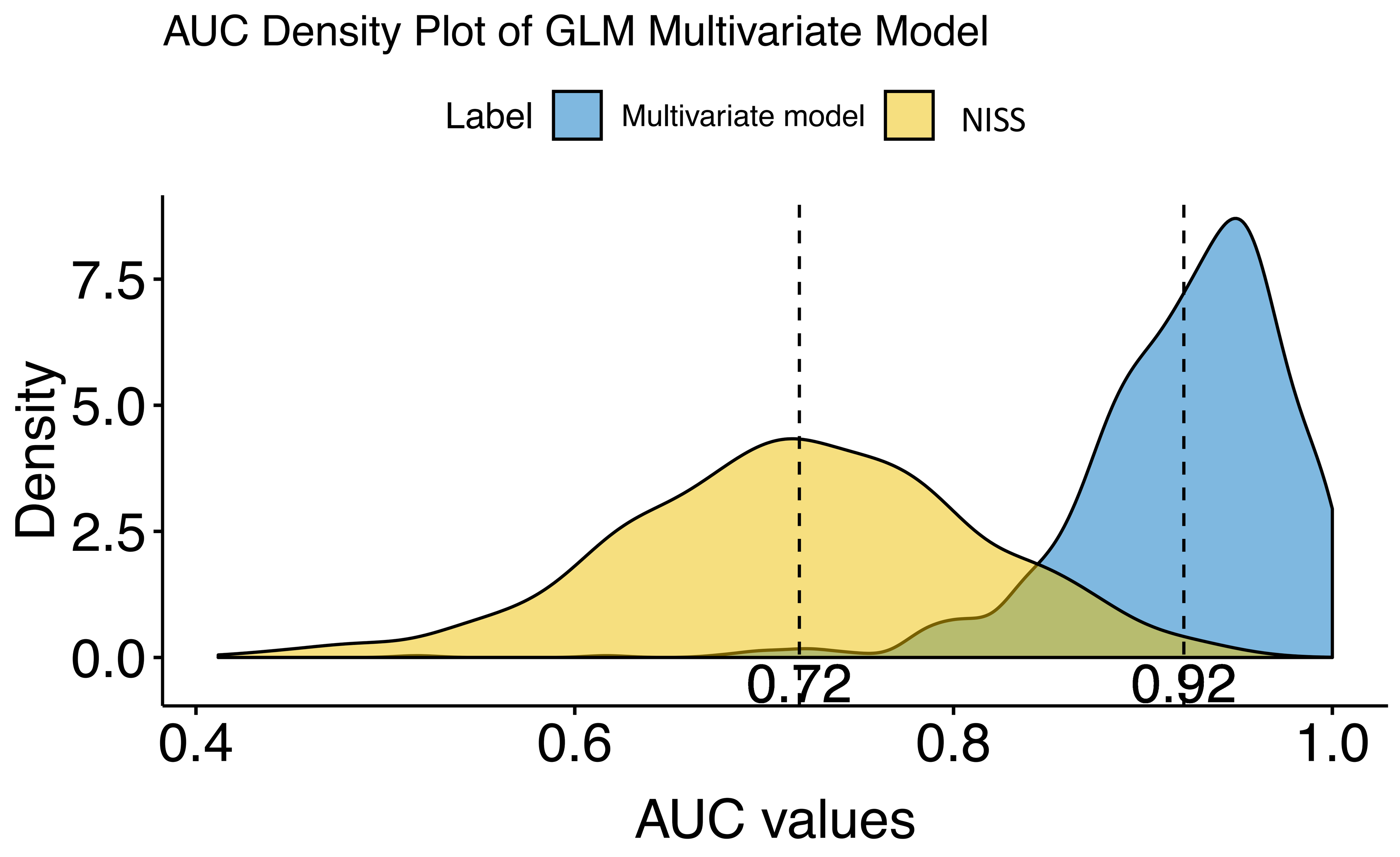

	Model AUC	Random Model AUC	p value
Multivariate	0.922 +/– 0.054	0.719 +/– 0.093	0.098

Supplementary Figure 2. **Comparison between AUC of multivariate model and the clinical variable alone:** The multivariate model (blue) consists of 3rd time point CD63 (MedFI) and decrease in CD62L with FMLf, first time point monocyte percentage and the clinical variable assessed (a. PS14, b. NISS). The clinical variable (yellow) is the performance of the feature by itself-. P-value was assessed through the overlap of the distributions.

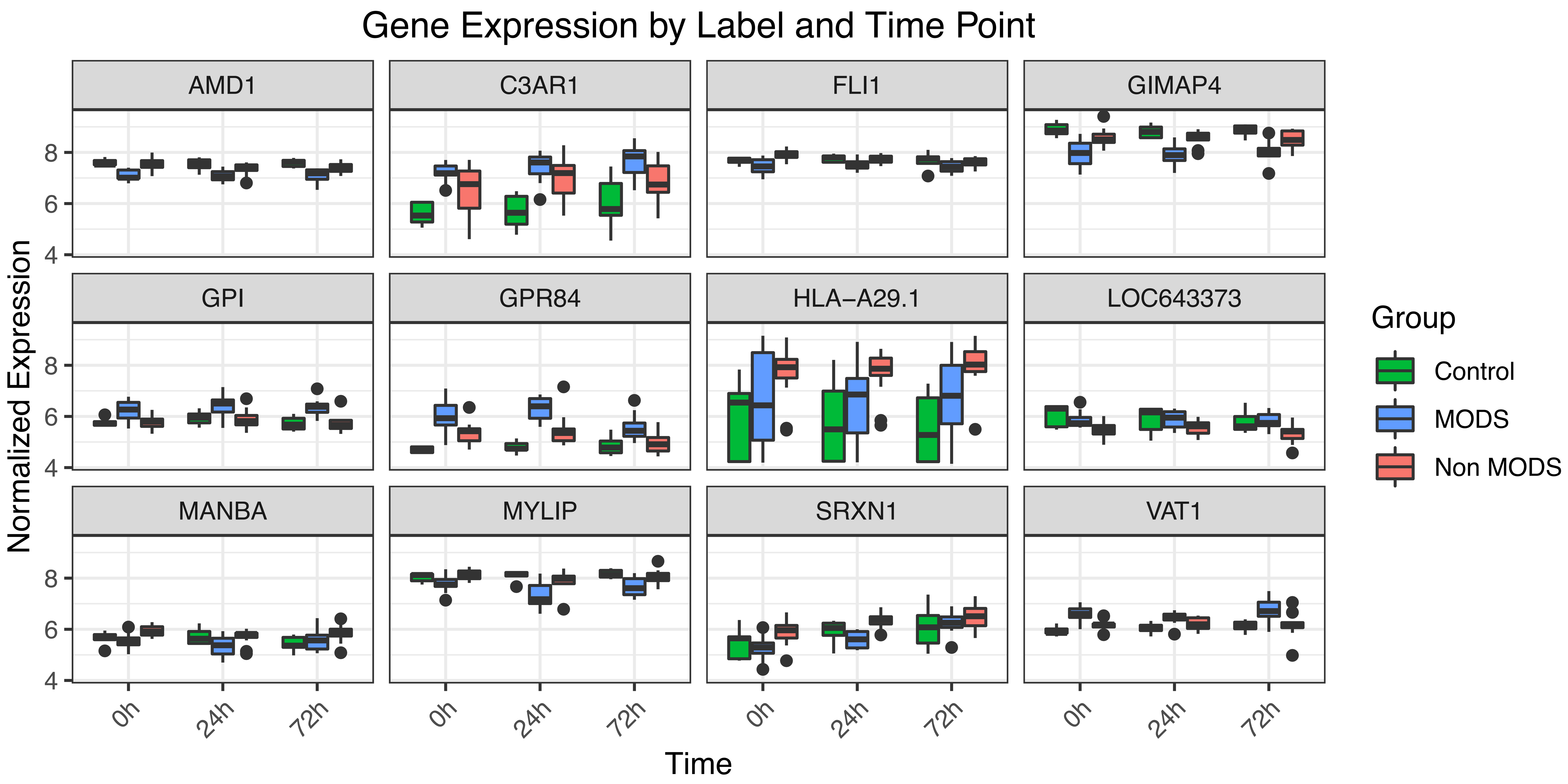

Supplementary Figure 3. **Genes selected through feature selection for all integrated time points (filter 50): Selected genes are:**

AMD1,C3AR1,FLI1,GIMAP4,GPI,GPR84,HLA.A29.1,LOC643373,MYLIP,VAT1,MANBA,SRXN1 with the code for the figure and gene expression data adapted from Cabrera et al [6]

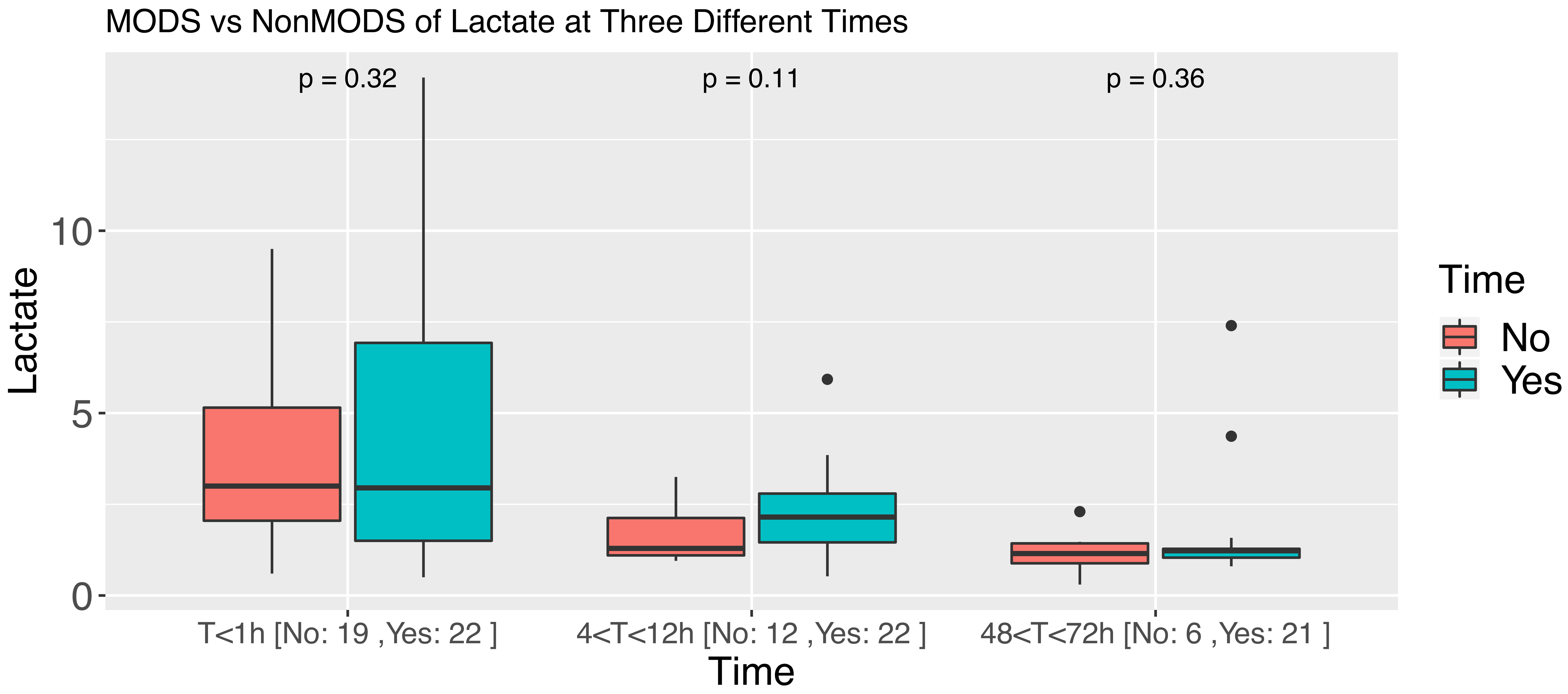

Supplementary Figure 4. **Lactate distribution analysis**. Boxplot of MODS and non MODS patients at the three different time points (<1h, 4 – 12 h ,48 – 72h). p-values calculated through unpaired t-test.
